# Therapeutic effect of hyperbaric oxygen in psoriasis vulgaris: two case reports and a review of the literature

**DOI:** 10.1186/1752-1947-0003-0000007023

**Published:** 2009-08-10

**Authors:** Glenn Butler, Julio Chávarri Michaels, Noori Al-Waili, Michael Finkelstein, Michael Allen, Richard Petrillo, Zev Carrey, Bangaruraju Kolanuvada, Bok Y Lee, Alfonso Gonzales Riera, Cesar Chávarri Michaels, Gary Urteaga

**Affiliations:** 1 Life Support Technologies, The Mount Vernon Hospital, Mount Vernon, NY, USA; 2 Department of Medicine, The Mount Vernon Hospital, South Shore Health System, Valley Stream, NY, USA; 3 Dr J Beale's Chronic Wound Management and Hyperbaric Center, The Mount Vernon Hospital, Mount Vernon, NY, USA; 4 Department of Surgery, New York Medical College, New York, USA; 5 Lima Center for Hyperbaric Medicine, Peru; 6 Corporación Hiperbárica Peruana, Peru

## Abstract

**Introduction:**

Psoriasis is an inflammatory and immunological cutaneous disease. The high morbidity in patients with psoriasis results from severe clinical manifestations and/or adverse effects of treatment. The Undersea and Hyperbaric Medical Society and Federal Medicare and Medicaid Services have approved the use of hyperbaric oxygen (HBO_2_) for more than 15 indications, including wound healing, infections and late effects of radiation, which are largely unresponsive to conventional treatments. Accumulated data show that HBO_2_ has anti-inflammatory effects and other positive influences on the immune system, making it a rational treatment in the management of psoriasis plaques and arthritis.

**Case presentation:**

We present the cases of two patients with long histories of psoriasis vulgarus who exhibited marked improvement with use of HBO_2._ The first patient was 40 years old and had pustular psoriasis and psoriatic arthritis. He was treated with six sessions of HBO_2_ (at 2.8 atmospheres of pressure for 60 minutes), which successfully controlled his symptoms. At the 18-month post-treatment follow up, the patient exhibited complete remission of psoriasis and marked improvement in psoriatic arthritis without medication. The second patient was 55 years old with extensive psoriatic lesions, and exhibited marked improvement within 15 sessions of HBO_2_. No adverse effects of HBO_2_ were identified.

**Conclusions:**

HBO_2_ may possess potential therapeutic efficacy in the management of psoriasis. We outline the pathogenesis of psoriasis and the selective anti-inflammatory and immunosuppressive effects of HBO_2_. We hope that this will provide a basis for elucidating the mechanisms of action and consequently pave the way for further controlled studies.

## Introduction

Psoriasis is a chronic, remitting and relapsing, immune-mediated inflammatory skin disorder with a strong genetic predisposition. It is among the most common immune-mediated diseases in humans, affecting 2.6% of the US population, and has significant social and economic impact. Current topical therapies used to manage psoriasis include steroids, vitamin D derivatives, retinoids, immunosuppressants, anthralin, coal tar ointment, and several other agents [1]-[5]. These drugs often have adverse effects that may be poorly tolerated. Light therapy includes ultraviolet B phototherapy or psoralen and ultraviolet A (PUVA) photochemotherapy. However, increased rates of nonmelanoma skin cancer have been observed following PUVA therapy [6]. Systemic therapies for psoriasis include methotrexate, cyclosporine, oral retinoids, and biologic therapies. A recent report reviewed the effectiveness and safety of the biologics alefacept, efalizumab, etanercept, and infliximab [7]. In addition to the reported adverse effects of the drugs, it was found that up to 40% of patients did not use their medication as directed.

Hyperbaric oxygen (HBO_2_) treatment is defined as breathing pure (100%) oxygen under conditions of increased atmospheric pressure. This results in elevated arterial oxygen tension to 2,000 mmHg or greater, which provides tissues with abundant oxygen. Possible complications of HBO_2_ therapy include barotrauma, oxygen toxicity (affecting the central nervous system and lungs), claustrophobia and anxiety, and ocular effects such as myopia and cataract. HBO_2_ promotes proliferation of fibroblasts, epithelial cells, and blood vessels in a wound. It can increase the killing ability of leukocytes and is lethal to certain anaerobic bacteria. Furthermore, it inhibits toxin formation by certain anaerobes, increases the flexibility of red cells, reduces tissue edema, and conserves intracellular ATP.

The Undersea and Hyperbaric Medical Society and the Federal Center for Medicare and Medicaid Services have approved the use of HBO_2_ in 14 indications including gas gangrene, necrotizing soft-tissue infections, diabetic foot ulcer, compromised grafts and flaps, bone infection, intracranial abscess, anemia and blood loss, crush injury, carbon monoxide and cyanide poisoning, radiation complications, decompression sickness, and gas embolism. HBO_2_ has potential effects on mediators of inflammation and the immune response. Our recent reviews [8,9] support the contention that HBO_2_ has anti-inflammatory and immunosuppressive properties. These properties make this treatment a potentially useful intervention that should be tested in the management of psoriasis and psoriatic arthritis.

## Case presentation

### Case 1

**Figure 1:**
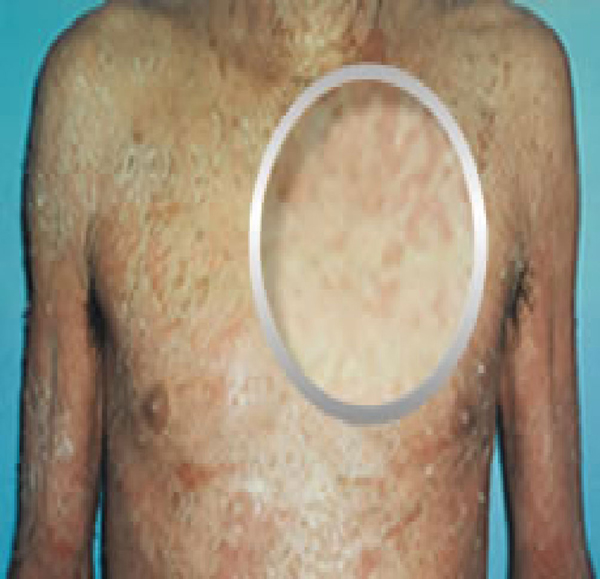
**Patient 1 before treatment with hyperbaric oxygen (Front View)**.

A 40-year-old man with disseminated erythrodermic psoriasis with pustules presented with arthralgias. He had a history of psoriasis vulgaris from infancy, diagnosed by skin biopsy, and had been followed by dermatologists. He had also had psoriatic arthritis since childhood. Physical examination revealed erythematous scaly plaques on his elbows, trunk and umbilical area, perineum and legs (Figure 1). He had interphalangeal distal joint involvement and spondylitis. His medical history was uneventful, with no evidence of underlying systemic disease and he was not taking any medications at the time of presentation.

**Figure 2:**
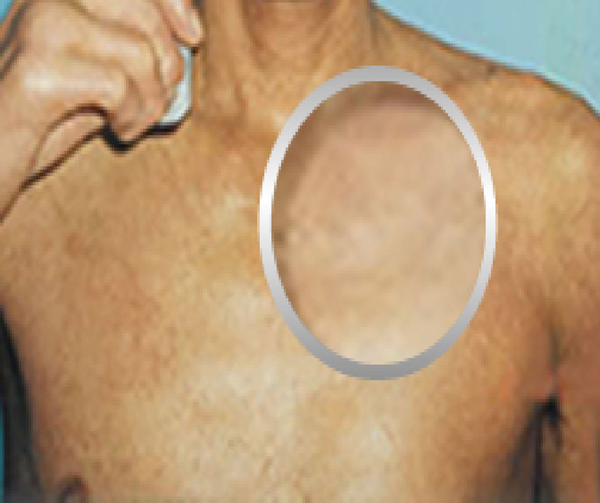
**Patient 1 after treatment with hyperbaric oxygen (Front View)**.

He requested a hyperbaric consultation after reading a newspaper article reporting on the use of HBO_2_ to treat psoriasis in a study conducted in Cuba. At the hospital he was evaluated to determine whether he was a suitable candidate for HBO_2_ therapy. After he had given informed consent, he underwent HBO_2_ therapy at 2.8 atmospheres for 60 minutes, once a day (5 days per week). The patient underwent a total of eight sessions, resulting in significant amelioration of symptoms (Figure 2). The patient did not receive any topical treatment before or during HBO_2_ therapy.

Most of the psoriatic lesions were cleared, with marked reduction in itching and scaling. No side effect was reported with the use of HBO_2_. The patient also reported less pain in his hands and joints after the eight sessions of HBO_2_. Follow up at 18 months revealed that the patient had only mild skin symptoms with degenerative changes of the arthritis.

### Case 2

**Figure 3:**
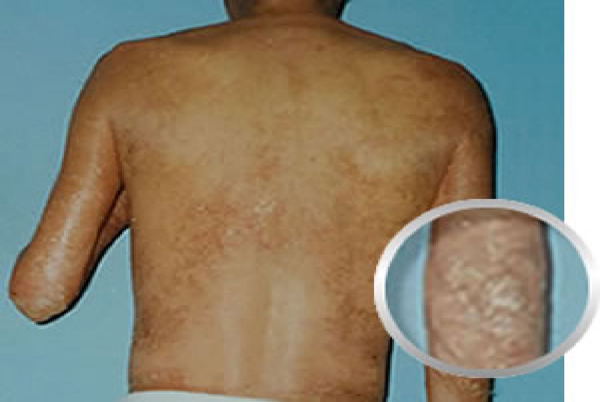
**Patient 1 before treatment with hyperbaric oxygen (Back View)**.

A 55-year-old man was referred for HBO_2_ for management of chronic bilateral leg ulcers and osteomyelitis. In addition, he had extensive psoriasis vulgaris (Figure 3). He had a long history of psoriasis vulgaris, which had been diagnosed by skin biopsy and followed by a dermatologist. His current medications at the time of admission to the hyperbaric department were topical mineral oil, Eucerin Calming Creme, and diphenhydramine 50 three times a day. He had erythema, and his skin was scaling and itching.

**Figure 4:**
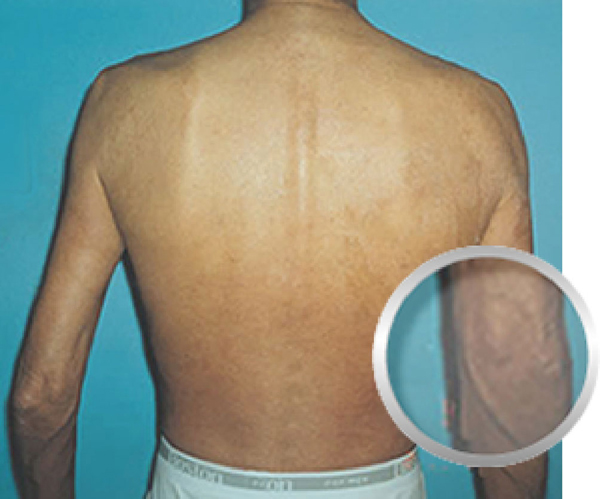
**Patient 1 after treatment with hyperbaric oxygen (Back View)**.

He underwent daily HBO_2_ at 2 atmospheres for 90 minutes, once a day (5 days per week). After six sessions his erythema, scaling and itching were reduced in severity, and after 15 sessions he had improved further (Figure 4). No adverse effect was reported with the use of HBO_2_.

## Discussion

The results presented here demonstrate the effectiveness of HBO_2_ in alleviating signs and symptoms of psoriasis in two patients. No adverse effects were reported during or after treatment with HBO_2_.

Leukocytes, cytokines, and keratinocyte growth or differentiation abnormalities are involved in psoriatic skin lesions. Psoriasis vulgaris is a T-cell-driven disease, with type I (interferon-γ-producing) T cells predominating in skin lesions [10,11]. A lymphocytic infiltrate in psoriasis plaques consists of a mixture of activated CD4^+^ and CD8^+^ T cells; the latter predominate in lesional epidermis and CD4^+^ cells in the dermis [12]. The therapeutic benefit of immunosuppressive drugs supports the view that activated T cells are pathogenic effectors of psoriasis [10]. Dendritic cells are found in psoriatic skin lesions, producing interleukin (IL)-12 and IL-23. Cytokine changes in psoriatic lesions consist of elevated levels of interferon-γ, tumor necrosis factor (TNF)-α, numerous interleukins (such as IL-1, IL-2, IL-6, IL-8, IL-12, IL-17, and IL-19), and multiple chemokines (MIG/CXCL9, IP-10/CXCL10, I-TAC/CXCL11, and MIP3α/CCL20) [11]. IL-12 p40 mRNA and expression of interferon-γ, inducible nitric oxide synthase, B7-1, and TNF-α are elevated in psoriatic tissue [11]. A rheumatoid-like pattern has been identified as one of the most common types of psoriatic arthritis. Autoantibodies directed against nuclear antigens, cytokeratins, epidermal keratins, and heat shock proteins have also been reported in psoriatic arthritis.

HBO_2_ suppresses the proliferation of macrophages and the formation of foam cells in atherosclerotic lesions [12]. HBO_2_ also intensifies the suppressive function of T lymphocytes, normalizes cell-bound immunity, and decreases the serum concentration in immune complexes [13]. The immunosuppressive effects of HBO_2_ include suppression of autoimmune symptoms, decreased production of IL-1 and CD4^+^ cells, and increased percentage and absolute number of CD8^+^ cells [9]. In addition, long-term HBO_2_ exposure suppresses development of autoimmune symptoms such as proteinuria, facial erythema, and lymphadenopathy. HBO_2_ decreases the CD4:CD8 ratio and proliferation of lymphocytes, and activates neutrophils to migrate to regions of high oxygen tension [14]. HBO_2_ suppresses TNF-α production induced by lipopolysaccharide, lipid A, and phytohemagglutinin A [15]. A marked decrease in IL-1 and IL-2 production, and a significant decrease in prostaglandin E_2_ production have been observed.

The positive clinical effects that HBO_2_ has in the treatment of chronic inflammation may relate to its effects on secretion of IL-1, IL-6, and TNF-α. The effects of HBO_2_ on prostaglandin, nitric oxide, and cytokines involved in wound pathophysiology and inflammation in particular were recently reviewed [8]. That review indicates that HBO_2_ has important effects on the biology of cytokines and other mediators of inflammation. HBO_2_ causes downregulation of cytokines and upregulation of growth factors. It transiently suppresses stimulus-induced proinflammatory cytokine production and affects the liberation of TNF-α and endothelins. Vascular endothelial growth factor levels are significantly increased with HBO_2_ therapy, whereas levels of prostaglandin E_2_ and cyclo-oxygenase-2 mRNA are markedly reduced. Therefore, the anti-inflammatory and immunosuppressive properties of HBO_2_ might account for its efficacy in the cases presented here.

**Figure 5:**
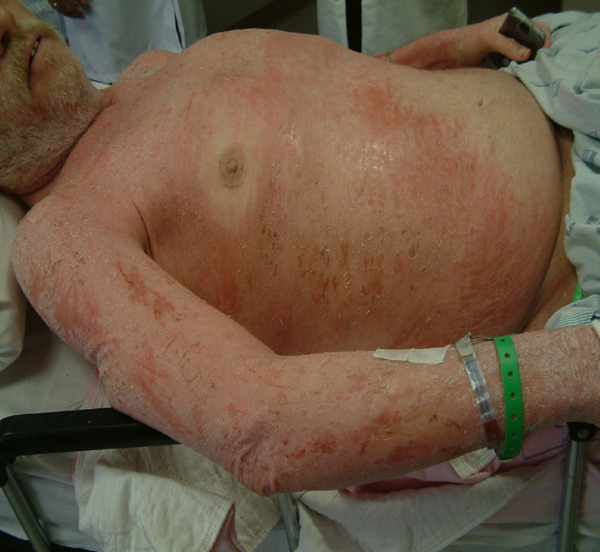
**Patient 2 before treatment with hyperbaric oxygen (Side View Torso)**.

**Figure 6:**
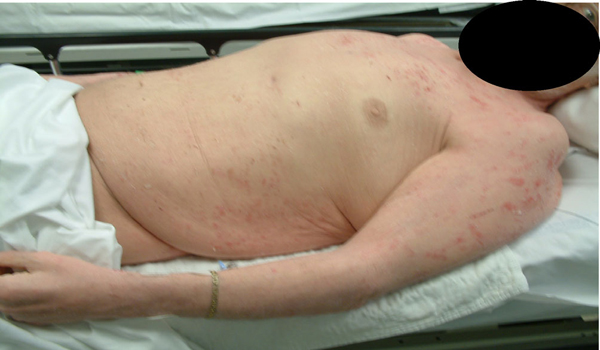
**Patient 2 after treatment with hyperbaric oxygen (Side View Torso)**.

**Figure 7:**
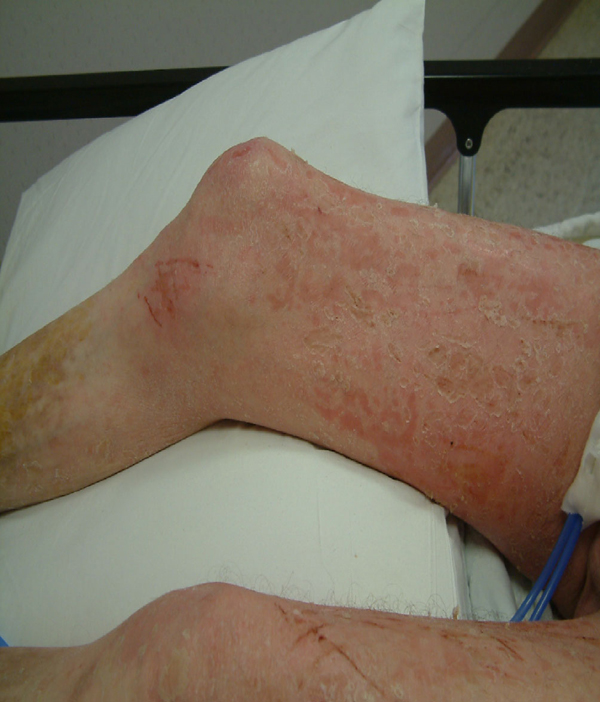
**Patient 2 before treatment with hyperbaric oxygen (Side View Legs)**.

**Figure 8:**
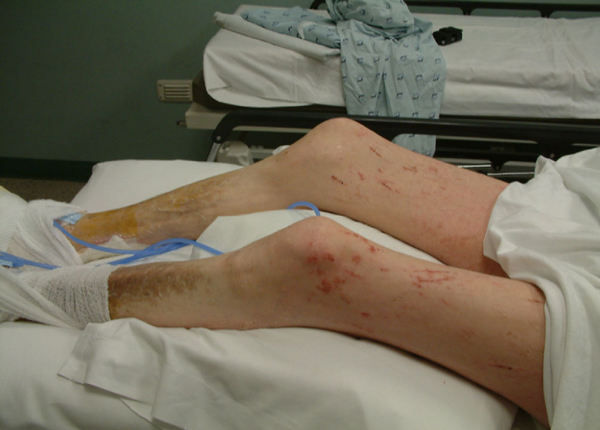
**Patient 2 after treatment with hyperbaric oxygen (Side View Legs)**.

## Conclusions

Our case reports, although suggestive, do not allow one to conclude that HBO_2_ treatment is useful in the treatment of psoriasis, because this condition can improve spontaneously. We emphasize that the findings presented here require confirmation by further controlled studies before a definitive conclusion may be drawn. We hope that our findings will also stimulate further investigation of the therapeutic potential of HBO_2_ alone or in combination with other modalities such as phototherapy in psoriasis.

HBO_2_ therapy may have a place in the management of psoriasis. Further studies including large numbers of patients and involving monitoring cytokines and inflammatory mediators will help us to explore the effect of hyperoxygenation on psoriasis and to elucidate its mechanism of action.

## Abbreviations

HBO_2_: hyperbaric oxygen; IL: interleukin; PUVA: psoralen and ultraviolet A; TNF: tumor necrosis factor.

## Competing interests

The authors declare that they have no competing interests.

## Consent

Written informed consent was obtained from the patient for publication of this case report and any accompanying images. A copy of the written consent is available for review by the Editor-in-Chief of this journal.

## Authors' contributions

GB conceived of the report and participated in its writing. NA-W conceived of the report, and conducted work and writing. RP, ZC, BK, MF, MA, and BL were involved in writing the Discussion. JM conceived of the report and conducted work. GU conceived of the report and conducted work. CM conceived of the report and conducted work. AR conceived of the report and conducted work. All authors read and approved the final manuscript.
